# Left Main Percutaneous Coronary Intervention and Transcatheter Aortic Valve Replacement in a Young Male with Rheumatic Heart Disease and Porcelain Aorta

**DOI:** 10.1155/2016/3671923

**Published:** 2016-06-22

**Authors:** Vinod Chainani, Osman Perez, Ram Hanno, Patrick Hourani, Pablo Rengifo-Moreno, Pedro Martinez-Clark, Carlos E. Alfonso

**Affiliations:** ^1^Department of Internal Medicine, Aventura Hospital and Medical Center, 20900 Biscayne Boulevard, Aventura, FL 33180, USA; ^2^Department of Internal Medicine, Texas Tech University Health Sciences Center Permian Basin Campus, 701 W. 5th Street, Odessa, TX 79763, USA; ^3^University of Miami, 1600 NW 10th Avenue No. 1140, Miami, FL 33136, USA

## Abstract

We highlight the presence of a calcified mass in the left main coronary artery without significant atherosclerosis seen in the other coronary arteries or in the peripheral large arteries. In our view, the calcified character of the obstruction and the calcification of the aortic valve are characteristic of a variant type of coronary artery disease (CAD) not associated with the same risk factors as diffuse coronary atherosclerosis, but, in this case, with rheumatic heart disease. This case report also emphasizes the interventional approach for patients with aortic valve stenosis secondary to rheumatic heart disease.

## 1. Introduction

Far from eradicated, after more than two hundred years since William Charles Wells linked acute rheumatic fever with carditis, rheumatic heart disease continues to be a disease of global proportions. In a recent extensive literature review by Carapetis and colleagues, it was estimated that there are over 15.6 million people affected by rheumatic heart disease globally with a reported 233,000 deaths annually [1]. Here we present the case of a young male with a variant type of coronary artery disease not associated with the typical risk factors for heart disease but with a prior history of rheumatic fever.

## 2. Case Presentation

A 57-year-old Caucasian male with history of rheumatic fever as a child, hypertension, and heavy alcohol use with associated cardiomyopathy presented with dyspnea on exertion for one year, worsening over the past two weeks. He had progressed from New York Heart Association Functional Classification (NYHA) Class II symptoms at baseline, including mild symptoms of shortness of breath during ordinary activities, to NYHA Class III, with marked limitation of activities during minimal exertion and was able only to feel comfortable at rest. He reported to have been previously evaluated at a different institution for similar but less severe symptoms three months prior to admission where he had an echocardiogram, the results of which are unknown. A cardiac catheterization was recommended but the patient left prior to the completion of this test.

**Figure 1:**
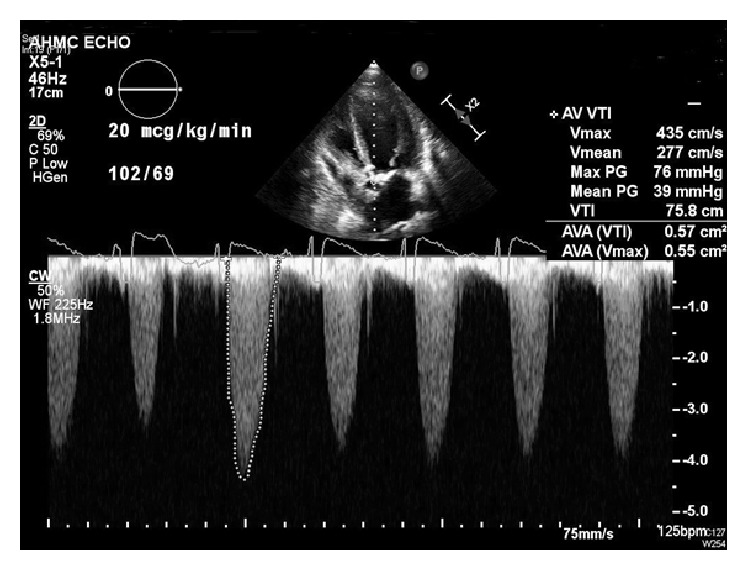
Dobutamine stress echocardiogram (DSE) at 20 mcg/kg/min of dobutamine. AVA: aortic valve area, HGen: midrange frequency, PG: pressure gradient, Vmax maximum velocity, Vmean: mean velocity, and VTI: velocity time integral.

**Figure 2:**
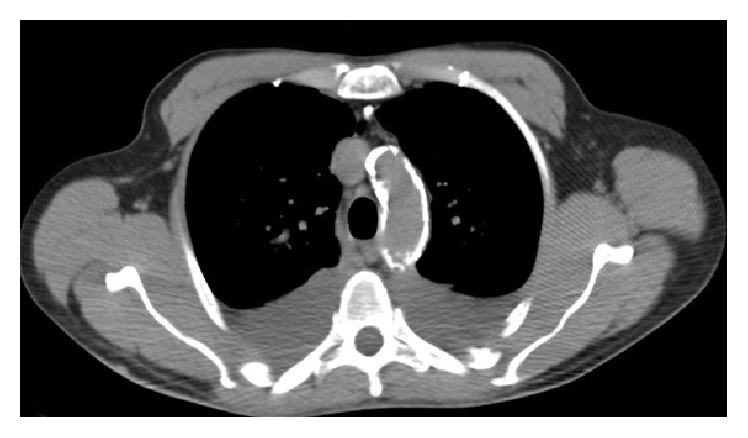
Chest computerized tomography, transverse plane.

Vital signs were within normal limits. A physical exam revealed the murmur of aortic stenosis. Echocardiogram showed a mean aortic valve (AV) gradient of 20 mmHg, an ejection fraction (EF) of 20%, and a calculated AV area of 0.61 cm^2^. A dobutamine stress echocardiogram (DSE) revealed a mean AV gradient of 39 mmHg at dobutamine 20 mcg/kg/min (43 mmHg at 15 mcg/kg/min), with a peak AV velocity of 435 cm/s, AV area of 0.55 cm^2^, and an EF of 37% (Figure 1). Coronary angiography revealed a calcified mass in the ostial left main coronary artery obstructing 80% of the vessel lumen. The chest computerized tomography (CT) showed a porcelain aorta without evidence of aneurysmal dilatation (Figure 2). No peripheral artery disease was visualized on CT or femoral angiography.

Due to the presence of porcelain aorta, a combination of coronary artery bypass grafting (CABG) and traditional surgical aortic valve replacement (SAVR) was not feasible. Alternatively, a left main percutaneous coronary intervention (PCI) was performed with Impella support. Patient was stabilized, medically optimized, and discharged home in stable condition to follow-up in two weeks for Transcatheter Aortic Valve Replacement (TAVR). Insurance issues complicated follow-up but, eventually, patient was successfully scheduled for TAVR four weeks after left heart catheterization and medical optimization as described above.

At the time of TAVR, initially the access was predilated and an E-sheath was advanced into the ascending aorta. Balloon aortic valvuloplasty was performed under rapid pacing at 180 beats per minute. The balloon was then exchanged for 26 mm Edwards XT. After positioning the bioprosthesis in the annulus of the native valve and under rapid pacing, the valve was deployed. The valve migrated towards the ventricle resulting in significant aortic regurgitation. Therefore, the decision was made to place a second valve, this time approximately 3 mm above the previous valve. A final aortogram showed evidence of trace aortic insufficiency; this finding was confirmed by echocardiography. A pigtail was advanced across the bioprosthesis into the left ventricle and measured no gradient across the aortic valve. Finally, the E-sheath was removed and the perclose sutures were tightened. Final digital subtraction angiogram showed no evidence of contrast extravasation.

## 3. Discussion

This case highlights the presence of a calcified mass in the left main coronary artery (LMCA) without significant atherosclerosis seen in the other coronary arteries or in the peripheral large arteries. The calcified character of the obstruction and the calcification of the aortic valve are characteristic of a variant type of CAD not associated with the same risk factors as diffuse coronary atherosclerosis, but, in this case, with rheumatic heart disease [2]. The role of inflammation without the presence of metabolic syndrome must be noted. The diffuse calcification of the aorta is part of an inflammatory condition and makes the need for nonsurgical intervention in this syndrome more likely.

Valve disease in rheumatic heart disease has been well described as sequelae of B- and T-cell activation in response to autoantigens in those with certain HLA subtypes [3]. Indeed, molecular mimicry among streptococcal M5 proteins, myosin, and valve-derived proteins causes T-cell cross reactivity leading to inflammation of myocardial tissue. The presence of chronic inflammation is evidenced by the presence of INF-*γ*, TNF-*α*, and IL-10 in biopsied valve tissue in patients with rheumatic heart disease [4]. Chronic inflammation likely perpetuated this patient's diffuse aortic and localized coronary calcifications ultimately requiring an otherwise appropriate CABG/SAVR candidate to undergo left coronary artery PCI and TAVR.

After an extensive literature research, including PubMed and Embase, on the use of TAVR for rheumatic aortic valve stenosis, we found only two articles presenting case reports on the topic. Bilge et al. presented two case reports of patients with rheumatic aortic stenosis and various comorbidities excluding them from open procedures. These patients ultimately underwent TAVR with a specific prosthetic valve [5]. Akujuo et al. presented a case in which TAVR was combined with transcatheter mitral valve implantation in a patient with both aortic and mitral valves affected by rheumatic valve stenosis [6].

The results of our literature search bolster the viability of TAVR for patient with rheumatic aortic stenosis while simultaneously highlighting the need for further trials including this patient population. As patients with rheumatic heart disease are usually of younger age and less complex comorbid state, they are usually classified as intermediate surgical risk and have classically been managed surgically. With the practical application of the recent PARTNER 2 trial results for intermediate surgical risk patients, we may see more use of TAVR in patients with rheumatic heart disease [7].
